# Pleiotropic ZIP8 A391T implicates abnormal manganese homeostasis in complex human disease

**DOI:** 10.1172/jci.insight.140978

**Published:** 2020-10-15

**Authors:** Laxmi Sunuwar, Azra Frkatović, Sodbo Sharapov, Qinchuan Wang, Heather M. Neu, Xinqun Wu, Talin Haritunians, Fengyi Wan, Sarah Michel, Shaoguang Wu, Mark Donowitz, Dermot McGovern, Gordan Lauc, Cynthia Sears, Joanna Melia

**Affiliations:** 1 Department of Medicine, Division of Gastroenterology and Hepatology, Johns Hopkins University School of Medicine, Baltimore, Maryland, USA.; 2 Genos Glycoscience Research Laboratory, Zagreb, Croatia.; 3 Laboratory of Glycogenomics, Institute of Cytology and Genetics of Siberian Branch of the Russian Academy of Sciences, Novosibirsk, Russia.; 4 Department of Medicine, Division of Cardiology, Johns Hopkins University School of Medicine, Baltimore, Maryland, USA.; 5 University of Maryland School of Pharmacy, University of Maryland, Baltimore, Maryland, USA.; 6 Department of Medicine, Division of Infectious Diseases, Johns Hopkins University School of Medicine, Baltimore, Maryland, USA.; 7 F. Widjaja Foundation Inflammatory Bowel and Immunobiology Research Institute, Cedars-Sinai Medical Center, Los Angeles, California, USA.; 8 Department of Biochemistry and Molecular Biology and; 9 Department of Molecular Microbiology and Immunology, Bloomberg School of Public Health, Johns Hopkins University, Baltimore, Maryland, USA.

**Keywords:** Gastroenterology, Genetics, Genetic variation, Glycobiology, Inflammatory bowel disease

## Abstract

ZIP8 is a metal transporter with a role in manganese (Mn) homeostasis. A common genetic variant in ZIP8 (rs13107325; A391T) ranks in the top 10 of pleiotropic SNPs identified in GWAS; A391T has associations with an increased risk of schizophrenia, obesity, Crohn’s disease, and reduced blood Mn. Here, we used CRISPR/*Cas9*-mediated knockin (KI) to generate a mouse model of ZIP8 A391T (Zip8 393T-KI mice). Recapitulating the SNP association with blood Mn, blood Mn was reduced in Zip8 393T-KI mice. There was restricted abnormal tissue Mn homeostasis, with decreases in liver and kidney Mn and a reciprocal increase in biliary Mn, providing in vivo evidence of hypomorphic Zip8 function. Upon challenge in a chemically induced colitis model, male Zip8 393T-KI mice exhibited enhanced disease susceptibility. ZIP8 391-Thr associated with reduced triantennary plasma N-glycan species in a population-based cohort to define a genotype-specific glycophenotype hypothesized to be linked to Mn-dependent glycosyltransferase activity. This glycophenotype was maintained in a cohort of patients with Crohn’s disease. These data and the pleiotropic disease associations with ZIP8 391-Thr suggest underappreciated roles of Mn homeostasis in complex human disease.

## Abstract 2

Abnormal manganese homeostasis is implicated by a GWAS disease-associated SNP, rs13107325 (ZIP8 A391T), studied in a knockin mouse model and human N-glycome analyses.

## Introduction

A nonsynonymous SNP in *SLC39A8* (rs13107325) is one of the most pleiotropic variants in the human genome, ranking ninth of 341 genomic regions associating with more than one human disease or trait in GWAS (1). In silico modeling predicts rs13107325 to be in the top 1.4% of deleterious substitutions in the human genome (2). The major allele associates with an increased risk of Parkinson’s disease, hypertension (3), and alcohol misuse disorders (4), and the minor allele associates with increased risk of schizophrenia (5), Crohn’s disease (6), obesity (7), dyslipidemia (8), and scoliosis (9). The minor allele frequency (MAF) is approximately 0.05 in American population and, 0.08 in Northern European populations and increases to 0.14–0.25 in the Ashkenazi Jewish population; the major allele is monomorphic in African and East and South Asian populations (10–12). Understanding how and why this pleiotropic SNP associates with disease offers the potential to uncover commonalities of pathogenesis and establish therapeutic strategies.

*SLC39A8* encodes ZIP8, a member of the *SLC39* family of transporters that increases cytoplasmic metals. The functional characterization of ZIP8 has been focused on induced zinc transport during inflammation (13–15); however, emerging data suggest a constitutive role for ZIP8 in manganese (Mn) homeostasis. In particular, individuals with private loss-of-function mutations in *SLC39A8* (distinct from rs13107325) have Mn deficiency and congenital disorders of glycosylation attributed to impaired activity of Mn-using glycosyltransferases (16–18). Zip8 inducible–knockout (iKO) mice have reductions in total Mn in liver, kidney, brain, and heart (19). Specific to ZIP8 391-Thr, a GWAS studying SNPs associated with blood metal levels found that ZIP8 391-Thr was the lead SNP associated with reduced blood Mn (9, 20), and small studies have found N-glycosylation defects in carriers of ZIP8 391-Thr (19, 21). Therefore, our hypothesis is that the pleiotropic effects of ZIP8 391-Thr could, at least in part, be driven by perturbations in Mn homeostasis and Mn-dependent processes, including N-glycosylation, with underrecognized clinical relevance in human disease.

To test this hypothesis, we used CRISPR/Cas9 in vivo gene editing to generate the Zip8 393T–knockin (Zip8 393T-KI) mouse taking advantage of the 84% homology between the human and mouse *Slc39a8* gene (10); mouse amino acid 393 corresponds with human amino acid 391 (Supplemental Figure 1; supplemental material available online with this article; https://doi.org/10.1172/jci.insight.140978DS1). At baseline, Zip8 393T-KI mice showed no anatomical abnormalities, despite abnormal Mn homeostasis, with reduced whole blood Mn (male and female mice), reduced liver and kidney Mn (male mice), and increased biliary Mn excretion (male and female mice). Both heterozygous and homozygous male KI mice showed enhanced susceptibility to epithelial injury in a chemical model of colitis, consistent with the SNP-disease association with Crohn’s disease (6); female KI mice did not exhibit a phenotype in the chemical model of colitis, suggesting possible sex-specific effects of Mn homeostasis and/or limitations of the dextran sodium sulfate (DSS) colitis model in female mice. Sex-stratified analysis of the genetic association between ZIP8 391-Thr and Crohn’s disease supported a stronger association in males. Finally, we demonstrated an association between the plasma N-glycan profile and ZIP8 391-Thr in the largest population-based study to date to our knowledge, specifically implicating reduced triantennary plasma N-glycan species. The accumulating human data, supported by the Zip8 393T-KI mouse model, support the relevance of abnormal Mn homeostasis and dysregulation of Mn-using processes, including N-glycosylation, in complex human disease.

## Results

### Zip8 393T-KI mice exhibit abnormal Mn homeostasis.

Regulation of Mn homeostasis in humans is an interplay between import and export proteins involved in enteric absorption, transport to the liver and other tissues, and excretion primarily via the hepatobiliary route (22). In the liver, Mn homeostasis is regulated by ZNT10 (also known as *SLC30A10*) at the apical membrane of hepatocytes, which excrete Mn (23); ZIP8 (*SLC39A8*) at the apical membrane of hepatocytes and bile canalicular cells, which reclaim Mn from bile (19); and ZIP14 (*SLC39A14*) at the basolateral membrane of hepatocytes, which mediate liver uptake of Mn from blood (24). Loss of function of ZNT10 and ZIP14 results in Mn excess (23, 24), whereas loss of function of ZIP8 leads to Mn deficiency (18, 19). In vitro data support that ZIP8 391-Thr results in hypomorphic function of ZIP8 (9, 25). We therefore hypothesized that Zip8 393T-KI mice would exhibit abnormal Mn homeostasis. It is well appreciated that Mn homeostasis exhibits age and sex specificity: males require more dietary Mn, absorb less dietary Mn, and have lower blood Mn levels compared with females (26, 27). We observed these trends in the Zip8 393T-KI mice and present data for both sexes.

**Figure 1:**
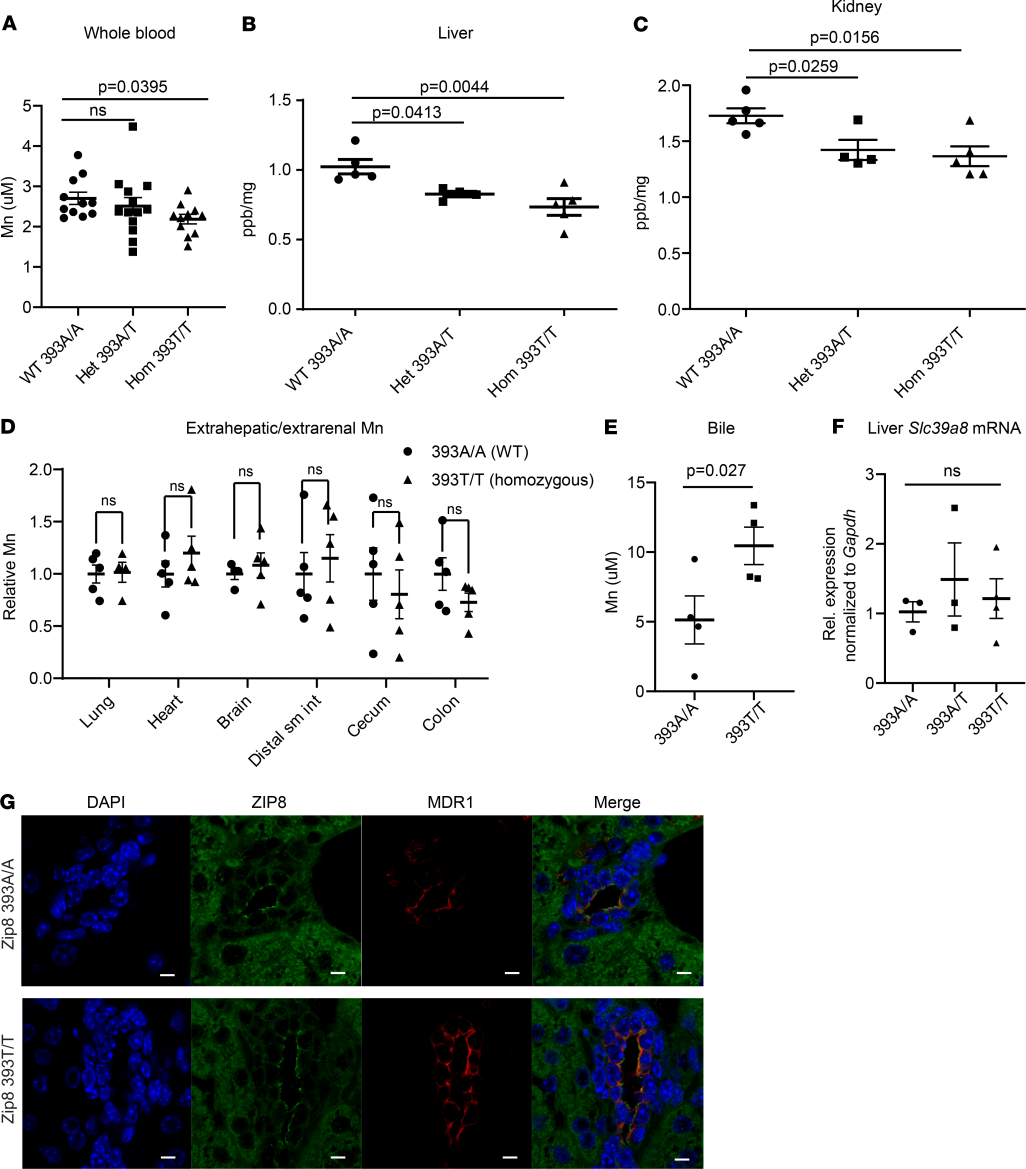
Zip8 393T-KI mice exhibit abnormal Mn homeostasis. (**A**) Whole blood Mn was reduced in Zip8 393T-KI homozygous mice (–18.9%, *P* = 0.0395). *n* = 11–13 male mice/genotype, 9–14 weeks of age, fasted. (**B**) Liver Mn was reduced in Zip8 393T-KI heterozygous and homozygous mice. (**C**) Kidney Mn was reduced in Zip8 393T-KI heterozygous and homozygous mice. (**D**) Relative Mn levels were comparable between WT and Zip8 393T-KI mice across additional tissue types. *n* = 3–7 male mice/genotype, 8–10 weeks of age (**B–D**). (**E**) Biliary Mn was increased in Zip8 393T-KI homozygous mice compared with WT mice. *n* = 4 male mice/genotype, 8–10 weeks of age, fasted. (**F**) There was no difference in liver *Slc39a8* mRNA across genotypes. Relative expression, normalized to *Gapdh* mRNA. *n* = 3 male mice/genotype, 8–10 weeks of age. (**G**) Zip8 localized to the bile canalicular membrane in a pattern similar to that of Mdr1 in WT and Zip8 393T-KI homozygous mice. Confocal images are representative of at least 3 animals/genotype. Scale bars: 5 μm. Whole blood (**A**) and bile Mn (**E**) were measured by atomic absorption spectroscopy; tissue Mn was measured by ICP-MS and normalized to wet tissue weight (**B–D**). Mean ± SEM. Statistical analysis by 1-way ANOVA with Kruskal-Wallis and Dunn’s multiple comparisons tests when 3 or more groups were compared (**A–C** and **F**); unpaired, 1-sided *t* test was used for comparison of 2 groups (**D** and **E**).

In male mice, whole blood Mn was reduced in the Zip8 393T-KI homozygous mice (–18.9%, *P* = 0.0395) compared with WT controls (Figure 1A), consistent with human data (20). To study systemic Mn homeostasis, we measured tissue abundance of select metals (see Methods). Liver Mn was reduced in the heterozygous (–19%, *P* = 0.0159) and homozygous (–28%, *P* = 0.0079) mice compared with that in littermate WT mice (Figure 1B). Kidney Mn was also reduced in heterozygous (–17.6%, *P* = 0.028) and homozygous (–21%, *P* = 0.033) mice (Figure 1C). Of note, tissue Mn was unchanged in brain, lung, heart, distal small intestine, cecum, and proximal colon (Figure 1D). These data are distinct compared with the Zip iKO mouse, where extrahepatic and extrarenal organs also exhibited relative reductions in Mn (19).

Additional findings relevant to known transport activities of ZIP8 included a trend toward reduced liver zinc, but not kidney zinc, in heterozygous and homozygous mice (*P* = 0.0502); there were no differences in plasma zinc levels (Supplemental Figure 2). Liver cadmium, but not kidney cadmium, was reduced in heterozygous (–25.0%, *P* = 0.0450) and homozygous mice (–27.3%, *P* = 0.0441) (Supplemental Figure 3, A and B). There were no differences in plasma or tissue iron (Supplemental Figure 3, C and D).

To test the hypothesis that hepatic Mn was reduced due to increased biliary losses (19), we measured biliary Mn. Consistent with the hypothesis, biliary Mn was increased 2-fold in Zip8 393T-KI homozygous mice compared with that in WT mice (*P* = 0.027) (Figure 1E). These findings are the most conclusive in vivo evidence that Zip8 393-Thr results in hypomorphic ZIP8 function as suggested by prior in vitro studies (9, 19).

Similar to that in the male KI mice, female Zip8 393T-KI homozygous mice exhibited reduced blood Mn and increased biliary Mn compared with WT mice. Liver Mn was reduced in female Zip8 393T-KI homozygous mice with nominal significance (*P* = 0.056). Notably, when comparing male and female mice of all genotypes, blood Mn was greater in the female mice, consistent with human data (22), while biliary Mn excretion was lower in the female mice compared with male mice (Supplemental Figure 4).

### Slc39a8 mRNA expression and Zip8 protein localization is unchanged in Zip8 393T-KI mice.

There are no reported expression quantitative trait loci associations between rs13107325 and any gene in GTEx (28). Most consistent with these data, there was no genotype-dependent effect on *Slc39a8* mRNA (Figure 1F). Zip8 localized to the apical membrane of bile canalicular cells, in a pattern consistent with Mdr1, independent of genotype (Figure 1G). These data suggest that ZIP8 391-Thr affects metal transport by mechanisms other than transcriptional regulation or protein localization.

### Male Zip8 393T-KI mice exhibit enhanced susceptibility to chemically induced colitis.

We next sought to challenge Zip8 393T-KI mice using a disease-relevant model to test if the Zip8 393T-KI mouse could recapitulate a disease phenotype implicated by the GWAS associations with rs13107325. Given the association with Crohn’s disease (6) and our previous work showing increased *SLC39A8* in active Crohn’s disease and expression in intestinal epithelial cells (25), we used a chemically induced colitis model with the hypothesis that KI mice would show enhanced susceptibility to intestinal epithelial injury. Because female mice exhibit relative resistance to DSS-induced injury, the experiments shown are in male mice (29); compared with the male mice, female mice of all genotypes were less susceptible to DSS-induced injury and failed to demonstrate a genotype-specific effect (Supplemental Figure 4).

**Figure 2:**
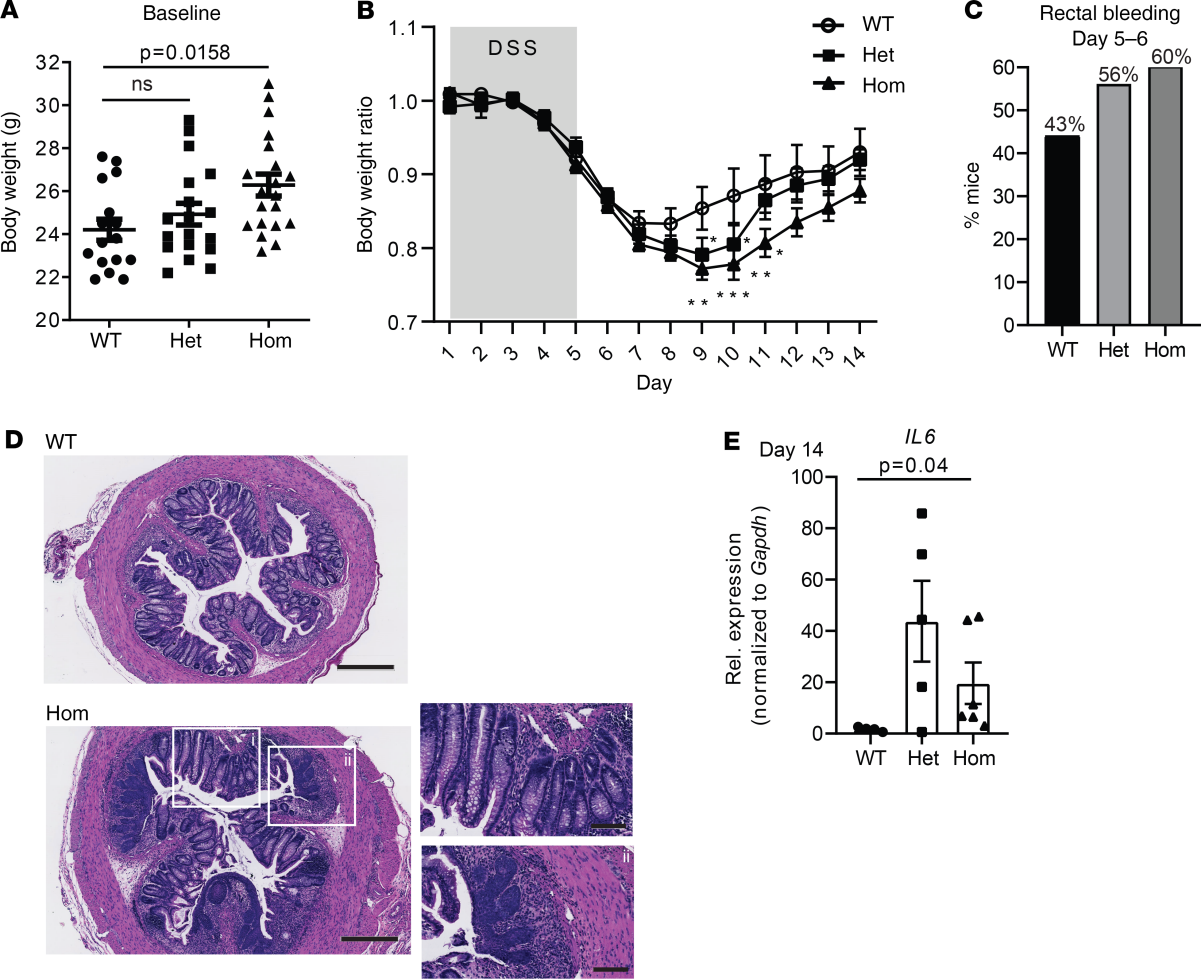
Zip8 393T-KI male mice exhibit increased susceptibility to chemically induced colitis. (**A**) Initial body weight of Zip8 393T-KI homozygous mice at 11.5–13 weeks of age was higher compared with that of WT and heterozygous mice. *n* = 16–20 male mice/genotype. (**B**) Zip8 393T-KI heterozygous and homozygous mice exhibited increased and more sustained weight loss in DSS-induced colitis model. Statistical analysis by linear regression (day 7–14). Slopes are equal between groups, but intercepts were significantly different between WT and Zip8 393T-KI heterozygous and homozygous mice (*P* < 0.0001), with no difference between Zip8 393T-KI heterozygous and homozygous mice (*P* = 0.9498). (**C**) Percentage of mice with rectal bleeding at day 5 and/or 6 was numerically higher in the heterozygous and homozygous mice (nonsignificant, χ^2^ test). (**D**) Day 14 histology was consistent with more inflammation in Zip8 393T-KI homozygous mice, with crypt elongation, crypt branching (top right), and expansion of lamina propria immune infiltrate and squamous metaplasia (bottom right). Scale bar: 400 μm; 200 μm (right). Images are representative of *n* = 5–7 mice/genotype. (**E**) Zip8 393T-KI heterozygous and homozygous mice had increased *Il6* mRNA, consistent with ongoing inflammation. Relative expression was graphed and normalized to *Gapdh* mRNA. *n* = 4–7 male mice/genotype. Statistical analysis by 1-way ANOVA with Kruskal-Wallis and Dunn’s multiple comparisons tests when 3 or more groups compared (**A** and **E**).

At baseline, mice of all genotypes (and sexes) showed no spontaneous intestinal inflammation. Male Zip8 393T-KI homozygous mice used for these experiments had higher baseline body weights when analyzed in aggregate compared with WT mice (Figure 2A). Mice were treated with 3.5% DSS for 5 days (Figure 2B). Weight loss in the WT mice nadired at day 7, while weight loss in the Zip8 393T-KI heterozygous and homozygous mice nadired at day 9, with a greater mean total percentage loss consistent with a more severe and sustained inflammatory response. Rectal bleeding developed in numerically more Zip8 393T-KI heterozygous and homozygous mice at days 5–6 (Figure 2C, nonsignificant, χ^2^ test). Histopathology analyses confirmed that Zip8 393T-KI heterozygous and homozygous mice exhibited more severe inflammation, extending into the muscularis mucosa, with epithelial cell loss (Figure 2D). Colonic *Il6* mRNA was elevated in the Zip8 393T-KI heterozygous and homozygous mice compared with WT mice, consistent with ongoing inflammation at day 14 (Figure 2E).

An expanded transcriptomic panel of immune-relevant genes was analyzed on bulk mRNA isolated from the distal colon for sham and day 8 and day 14 treated WT and homozygous mice to study possible genotype-specific alterations to the mucosal immune microenvironment. These data are limited after adjustment for multiple comparison testing, but, as exploratory data, the most significantly differentially expressed genes at day 8 were protein kinase C α (*Prkca*) (log_2_ fold decrease of –1.35) and *Il11* (log_2_ fold increase of 5.71) in homozygous Zip8 393T-KI mice compared with WT mice (Supplemental Table 1). *Prkca* is a Th17 cell–selective kinase regulating IL-17A production (30) and acts as a tumor suppressor in the intestine (31); reduced expression is associated with other immune-mediated phenotypes, including multiple sclerosis (32). Mouse models support IL-11 as a profibrotic cytokine (33, 34). Recent single-cell RNA-Seq data have shown an association between inflammatory-type fibroblasts (IL13RA2^+^-IL11^+^), IL-11, and Crohn’s disease (35) and ulcerative colitis (36). Given the specific association of ZIP8 391-Thr with stricturing and penetrating Crohn’s disease (6), the enhanced expression of a profibrotic cytokine is of particular interest; however, this will require further confirmatory mouse and human studies.

### Human genetic association between ZIP8 391-Thr and Crohn’s disease is more significant in male mice than in female mice.

The enhanced susceptibility to DSS-induced injury in male KI mice compared with female KI mice prompted us to ask if the association between ZIP8 391-Thr and Crohn’s disease exhibits a sex-specific signal. Sex-stratified analysis was performed in a Crohn’s disease case-control cohort (female, *n* = 7378; male, *n* = 7927). There is an association between ZIP8 391-Thr and Crohn’s disease in both sexes; however, notably, the association is more significant in males (*P* = 4.15 × 10^–9^) than females (*P* = 1.93 × 10^–5^) (Supplemental Table 2). It is possible that the results are biased toward greater significance in males because the cohort includes more male individuals. However, we also speculate that these data may support differential, sex-specific effects of ZIP8 391-Thr in Crohn’s disease and reinforce the role and impact of sex-specific mechanisms in Mn homeostasis.

### ZIP8 391-Thr is associated with reduced plasma triantennary N-glycans but not aberrant IgG N-glycosylation in human population-based samples.

Finally, we sought to reinforce the relevance of abnormal Mn homeostasis associated with ZIP8 391-Thr in human data. Two small cohort studies have shown an association between ZIP8 391-Thr homozygous carriers and increased abundance of monosialo-monogalacto-biantennary plasma glycans hypothesized to be secondary to reduced activity of Mn-dependent β-1,4-galactosyltransferase (19, 21). Population-based studies of the plasma N-glycome were needed. Further, given the effect of alternative N-glycosylation at the Asn297 site in the Fc domain of IgG on antibody effector function (37) and the initial observations of hypogalactosylation associated with ZIP8 391-Thr (19, 21), we hypothesized that there would be an association between ZIP8 391-Thr and N-glycosylation of IgG. To study associations between ZIP8 391-Thr and the N-glycome, we used GWAS of human blood plasma (*n* = 2763) (38) and IgG N-glycome (*n* = 8080) (39), where N-glycome profiles were analyzed using ultra-high-performance liquid chromatography (UHPLC). The frequency of ZIP8 391-Thr was 8% and 8.3%, respectively; notably, ZIP8 391-Thr (rs13107325) was not included in the initial analyses, as it was not included on the original genotyping arrays. In these cohorts, ZIP8 391-Thr was associated with reduced plasma triantennary traits, including trisyalated and trigalactosylated structures (Table 1 and Supplemental Table 3). However, there were no associations between ZIP8 391-Thr and N-glycosylation of IgG (Supplemental Table 3).

### The ZIP8 391-Thr–associated plasma N-glycan profile defines a distinct glycophenotype in patients with Crohn’s disease.

Plasma and IgG N-glycan profiles of patients with inflammatory bowel disease (IBD) have been recently studied by UHPLC (40, 41). Increased triantennary and tetra-antennary glycan species are features of the IBD-associated plasma N-glycome compared with healthy individuals; therefore, we next studied whether there was an interaction between ZIP8 391-Thr and the plasma and IgG N-glycome in a cohort of patients with Crohn’s disease (*n* = 313). The frequency of ZIP8 391-Thr was 18% (6). Consistent with the genotype-specific plasma N-glycan profile found in the population data, 3 triantennary glycan species were decreased in patients with Crohn’s disease with ZIP8 391-Thr compared with noncarriers (Table 2 and Supplemental Table 4), and there were no associations with IgG N-glycosylation. These data are distinct from the published analysis of patients with Crohn’s disease (agnostic to genotype) compared with controls, where there were no differences in 2 of the glycan species (A3F0GE and A3GE), and H6N5E3 was increased (40). Therefore, the ZIP8 391-Thr–associated glycophenotype defines a distinct patient subset within Crohn’s disease.

## Discussion

rs13107325 (ZIP8 A391T) is one of the most pleiotropic SNPs in the human genome, and approximately 5%–10% of individuals in some human populations carry this SNP. Taking advantage of the conserved sequence homology of ZIP8 and in vivo CRISPR/*Cas9* gene editing to KI the disease-associated variant, we have generated an animal model that demonstrates that ZIP8 391-Thr results in abnormal Mn homeostasis, with reduced liver and kidney Mn, reduced blood Mn, and elevated biliary Mn excretion. Under inflammatory challenge in the DSS-induced colitis model, male Zip8 393T-KI mice were more susceptible to DSS-induced injury, mimicking the association between ZIP8 391-Thr and Crohn’s disease. One measurable impact of abnormal Mn homeostasis in humans is reinforced by the SNP-N-glycome analyses, which found an association between ZIP8 391-Thr and reduced triantennary N-glycans in plasma, with no associated N-glycan perturbations of IgG. The sum of the human and murine data suggests that pathology (and perhaps a yet-to-be-understood survival benefit that has kept ZIP8 391-Thr under positive selection; ref. 10) is driven, at least in part, by hypomorphic activity of ZIP8 and abnormal Mn homeostasis.

In addition to providing in vivo evidence of hypomorphic Zip8 function in the setting of 393T-KI, the Zip8 393T-KI mouse model importantly exhibits a more limited distribution of tissue sites, with relative reductions in Mn compared with the inducible ZIP8-knockout mouse model (19). The 2 mouse models were not tested in parallel in this report, yet comparing our findings against the published literature (19) (Supplemental Table 5) shows that male Zip8 iKO and liver-specific knockout results in a 69%–77% reduction in liver Mn compared with that in matched controls, while Zip8 393-TI KI male mice exhibited a 19% (heterozygous) and 28% (homozygous) reduction in liver Mn. Whole blood Mn is only reported in the Zip8 liver-specific knockout mouse with a 65% reduction of whole blood Mn compared with that of WT mice (19), whereas we observe a 18.9% reduction in the homozygous Zip8 393T-KI male mice. Therefore, we estimated the function of Zip8 in the context of Zip8 393T-KI to be approximately 70% compared with the WT allele, and because heterozygous Zip8 393T-KI mice also exhibited relative, albeit smaller reductions, in liver and kidney Mn, we speculated that Zip8 393T may act as a dominant negative mutation, although this remains to be tested.

The limited tissue distribution of relative Mn reductions in the Zip8 393T-KI model compared with the Zip8 iKO model underscores an incomplete understanding of possible compensatory mechanisms at play when ZIP8 function is perturbed. As demonstrated by the lethality of Zip8 global knockout mouse models (42, 43), Zip8 is required for embryogenesis and early life. Understanding potential interactions among ZIP8, ZIP14, and ZNT10 (as key regulators of Mn homeostasis) (44) or other compensatory mechanisms will require dedicated study across the mouse models. The additional variable of dietary Mn will also require testing. Together, these experiments will afford a more complete appreciation of the biological impact of ZIP8 391-Thr, mechanisms of Mn homeostasis, and interaction with dietary Mn.

The DSS colitis model experiments provide important evidence that male Zip8 393T-KI homozygous and heterozygous mice, although phenotypically indistinguishable from WT mice at baseline, exhibited a disease-relevant phenotype once challenged. We acknowledge that the relevance of the DSS colitis model to ileal Crohn’s disease, the subphenotype of Crohn’s disease with the strongest association with ZIP8 391-Thr (6), can be debated; yet with this limitation, we use DSS colitis as a challenge of intestinal homeostasis in the setting of intestinal injury. Both the heterozygous and homozygous Zip8 393T-KI male mice exhibited a more severe phenotype than WT mice. We note a recent report of enhanced susceptibility to DSS-induced injury in mice fed Mn-deficient chow (45). We acknowledge that our experiments do not directly test that abnormal Mn homeostasis specifically drives the colitis phenotype, and future experiments will test variable Mn dietary content. Impairment of other activities of ZIP8, such as zinc-mediated regulation of NF-κB signaling (14) or the zinc/ZIP8/MTF1 transcriptional cascade (15), may participate. The effect of inflammation on Mn homeostasis has not been tested. Finally, the physiologic effects of increased biliary Mn are unknown but may have particular importance when considering how dysregulation of Mn in the liver affects the gut: a single study in a rodent model supports an interaction between increased taurohyodeoxycholic acid and low biliary Mn (46); however, differential bile salt composition in the context of elevated biliary Mn has not been reported.

Coupled with understanding of the role of Mn homeostasis on the colitis phenotype in the Zip8 393T-KI mice is study of the underlying sex-specific mechanisms of Mn handling and why the female Zip8 393T-KI mice failed to show a phenotype. Human studies have shown that blood Mn is reduced in males compared with females (22), a phenotype recapitulated in the Zip8 393T-KI mouse model. Further, males are known to require more dietary Mn than females (22). To our knowledge, sex-specific differential biliary Mn levels have not been previously explored. Is this sexual dimorphism with biological relevance linked to the sex-specific Mn homeostasis? Is the lack of phenotype in the female mice using the DSS-induced colitis model related to an inherent limitation of the DSS model (29) or, again, sex-specific Mn homeostasis? Are females more tolerant of Mn dyshomeostasis? Systematic assessment of the absorption, distribution, and excretion of radiolabeled Mn; characterization of the mucosal immune microenvironment; and additional models of intestinal inflammation, such as bacterial infection models, may provide useful approaches to study these questions. Further, there are recent gene-interaction studies in children examining neurodevelopment and polymorphisms in Mn transporters, including ZIP8 391-Thr; these studies reinforce that genotype, sex, age, and timing of Mn exposures influence phenotype (47, 48), therefore these factors will require study. We put forward the Zip8 393-Thr KI mice to provide a critical model with direct human relevance for further such studies.

The SNP-N-glycome analyses clarify that ZIP8 391-Thr–associated abnormal Mn homeostasis associates with reduced triantennary N-glycan branching in plasma (but not IgG N-glycome species) and implicates Mn-using, N-acetylglucosaminyltransferases and/or downstream Mn-using galactosyltransferases and sialotransferases. We speculate that the reduced abundance of trisylated and trigalactosylated glycans may be secondary to a critical limitation of Golgi N-acetylglucosaminyltransferases –— our data implicate N-acetylglucosaminyltransferase IV (MGAT4), which catalyzes the formation of triantennary glycans. In support of this hypothesis and because all N-glycan species on IgG are biantennary, bigalactosylated, and bisyalated in their most complex form (49), we found no association between ZIP8 391-Thr and the IgG N-glycome. Finally, if generation of triantennary glycans is impaired, we would expect reduced abundance of downstream tetra-antennary glycans. We found a trend in this direction; however, it did not meet statistical significance after FDR correction. This may reflect the overall low abundance of tetra-antennary glycans in plasma (<5.6% of total glycosylated proteins) impairing the power to detect genotype-dependent differential abundance (50).

Clinically, the association between ZIP8 391-Thr and reduced triantennary N-glycans begs the question, what is the significance of the ZIP8 391-Thr glycophenotype? α-1-Acid glycoprotein (AGP) is the most abundant plasma protein with triantennary and tetra-antennary N-glycans (50) and the leading protein to prioritize for study of the ZIP8 391-Thr glycophenotype. AGP is an acute-phase reactant described as an immunomodulator and a major binding protein for endogenous ligands and drugs (51). AGP has 5 predicted N-glycosylation sites, with over 150 glycoforms that affect the AGP-ligand-binding site and change protein dynamics (52, 53). We speculate that further study of the differential biological function of AGP glycoforms, driven in part by ZIP8 391-Thr genotype and abnormal Mn homeostasis, may reveal mechanistic underpinnings of the pleiotropic disease associations of ZIP8 391-Thr. We acknowledge that a limitation to our current work is the failure to demonstrate that the Zip8 393T-KI mice recapitulate the human glycophenotype; these studies are underway.

Mn status is not routinely considered in clinical practice; however, we position our data to postulate that abnormal Mn homeostasis and dysregulation of Mn-dependent processes have underappreciated clinical implications — potentially with sex-specific effects. This central hypothesis will require further translational studies to understand how abnormal Mn homeostasis and the effect on Mn-using pathways (such as N-glycosylation) contribute to the pathogenesis of the diverse set of human diseases associated with ZIP8 391-Thr.

## Methods

### Generation of the Zip8 393T-KI mouse.

We designed 2 single-guide RNA targeting Zip8 393Ala and a single-strand donor oligonucleotide for homology-directed repair harboring the 3 point mutations to KI our allele of interest, mutate the PAM site, and insert a restriction enzyme site for genotyping (Supplemental Figure 1A). Pronuclear injection of 1-cell C57BL/6J embryos (The Jackson Laboratory) was performed by the Johns Hopkins University Transgenic Core with standard microinjection techniques (54) using a mix of Cas9 protein (30 ng/μL, PNABio), tracrRNA (0.6 μM, Dharmacon), crRNA (0.6 μM, IDT), and ssDNA oligo (10 ng/μL, IDT) diluted in RNase-free injection buffer (10 mM Tris-HCl, pH 7.4, 0.25 mM EDTA). Injected embryos were transferred into the oviducts of pseudopregnant ICR female mice (Envigo) using the technique described in Nagy et al. (55). Of 29 pups obtained, 5 had the desired modified alleles. These founder male mice were bred to Jackson B6 female mice to generate the heterozygous F2 generation that was used for breeding thereafter. Genotyping was accomplished using a nested PCR approach and restriction enzyme digestion (Supplemental Figure 1B) and confirmed by Sanger sequencing (Supplemental Figure 1C). Heterozygous mating yielded pups following an expected Mendelian genetics distribution. Mice were fed a standard mouse chow (Harlan Teklad Global 18% Protein Extruded Diet 2018SX, 100 pm Mn); of note, 100 ppm Mn is considered a Mn supplemented diet, whereas Mn “replete diets” most commonly include only 10 ppm Mn.

### Inductively coupled plasma mass spectrometry.

Tissues were harvested promptly after sacrifice, placed in metal-free conical tubes (VWR), and wet weight was recorded. To each sample, 200 μL nitric acid (67%–70% [w/w], TraceMetal Grade) was added, and the samples were incubated at 80°C for 18 hours. The samples were then removed from the oven and diluted with 2 mL milliQ water to achieve a final nitric acid concentration of 6%. Metal concentrations were determined using Agilent 7700× inductively coupled plasma mass spectrometry (ICP-MS) (Agilent Technologies) with an Octopole Reaction System cell in He mode to remove any interferences. The ICP-MS parameters used for the metal analysis were as follows: an RF power of 1550 W, an argon carrier gas flow of 0.99 L/min, helium gas flow of 4.3 mL/min, octopole RF of 200 V, and OctP bias of –18 V. Samples were directly infused using the 7700× peristaltic pump and a micromist nebulizer. Metal concentrations for arsenic, calcium, chromium, copper, cadmium, iron, magnesium, Mn, nickel, and zinc were derived from a calibration curve generated by a series of dilutions of metal atomic absorption standard (Fluka Analytical) prepared in the same matrix (6% nitric acid) as the samples. Data analysis was performed using Agilent’s Mass Hunter software.

### Atomic absorption spectroscopy.

Metal analysis of whole blood and bile was carried out by atomic absorption spectroscopy (AAS) performed on a PerkinElmer Life Sciences AAnalyst 600 graphite furnace atomic absorption spectrometer. For whole blood assay, the blood was digested with nitric acid (1:2) (Fisher Chemical, TraceMetal Grade) and further diluted in double distilled water for AAS.

### Immunofluorescence.

Paraffin-embedded tissue sections were deparaffinized, rehydrated, and blocked with 10% NGS and 5% BSA for 1 hour after antigen retrieval with EDTA (1 mM at pH 8) (100^o^C for 30 minutes). The sections were washed with TBST and incubated with anti-ZIP8 (MilliporeSigma, HPA038832) (1:50), MDR1 (Santa Cruz), and E-cadherin (1:100) (BD Transduction Laboratories) overnight at 4°C. The following day, sections were washed with TBST and stained for 1 hour at room temperature with anti-rabbit Alexa Fluor 488 (Life Technologies) (1:100) and anti-mouse Alexa Fluor 568 as the secondary antibodies, while they were protected from light and exposed to counterstains for DAPI. The sections were imaged on the Olympus FV3000RS confocal microscope with the ×40 oil immersion objective as indicated.

### mRNA extraction and quantitative PCR.

RNA was extracted using the QIAGEN RNeasy Mini Kit per the manufacturer’s protocols. Purified RNA concentrations were measured using wavelengths of 260/280 nm on the Take3 micro-volume plate system (Biotek). From equal amounts of RNA, cDNA was generated using iSCRIPT. Real-time quantitative PCR was performed and analyzed using SYBRGreen reagents on the QuantStudio 12K flex platform. Primer sets used are included in Supplemental Table 6; *Gapdh* was included as an internal control, and relative expression was calculated using the 2(–delta delta Ct) method.

### DSS-induced colitis.

Mice of all 3 genotypes were cohoused for the experiment. Mice at 11.5–13 weeks of age were fed 3.5% (w/v) DSS (MP Biomedicals; MW = 36,000–50,000) dissolved in sterile water ab libitum for 5 days. The 3.5% DSS water was replenished after 2 days and continued until the fifth day. After 5 days, the mice were switched to sterile drinking water and followed for up to 9 days. The mice were monitored daily to assess survival, weight loss, and presence of rectal bleeding. At the end of the experiment, the mice were sacrificed by CO_2_ inhalation and terminal bleeding. Body weight as well as colon, liver, and spleen weight and colon length were recorded. The cecum and colon as a “swiss roll” were fixed in formalin and prepared for histology. The distal 1 cm of colon was reserved for mRNA.

### Distal colon RNA transcriptome analysis.

mRNA was isolated as above from distal colon sections from sham- and DSS-treated (day 8 and day 14) WT and male homozygous Zip8 393T-KI mice. Transcriptomic analysis was performed on the nCounter Mouse Inflammation V2 panel (Nanostring Technologies), consisting of 248 genes and 6 housekeeping genes according to the manufacturer’s protocol. Raw reads were normalized by background thresholding, geometric mean of positive controls, and code set content normalization with housekeeping genes. Data analysis was performed using R (v3.3.2) and NanoString Advanced Analysis platform for differential gene expression.

### Sex-stratified analysis of association between rs13107325 and Crohn’s disease.

rs13107325 genotyping was available from the Illumina HumanExome+ array as previously described (6). Sex-stratified logistic regression, adjusting for population substratification with 4 principal components, was performed to determine strength of association with Crohn’s disease and direction of effect in each sex separately (PLINK v1.9) (56, 57). Sex-combined regression was performed for comparison.

### Secondary analyses of SNP-N-glycome associations.

To explore the association between ZIP8 391-Thr and human N-glycome in human population-based samples, we used the results of recently published GWAS of human blood plasma N-glycome (*n* = 2763) (http://doi.org/10.5281/zenodo.1298406) (38) and IgG N-glycome (*n* = 8080) (https://datashare.is.ed.ac.uk/handle/10283/3238) (39), where N-glycome profiles were analyzed using UHPLC. We performed a single-SNP glycome-wide association analysis among 113 total plasma N-glycome traits and 77 IgG N-glycome traits using publicly available GWAS summary statistics. The association statistics were extracted and FDR was applied to correct for the multiple testing. The significance threshold for the SNP-N-glycome association was determined by correcting for the principal components (30 + 21) that explain 99% of variance in 113 tested plasma traits and 99% of variance in 77 tested IgG traits (*P* ≤ 0.05 / [30 + 21] = 9.8 × 10^–4^).

Sex- and age-corrected total plasma (*n* = 141 traits) and IgG (*n* = 81 traits) N-glycomes for patients with IBD were measured or derived as previously published (40, 41). rs13107325 genotyping was available from the HumanExome+ array (Illumina) (6) or TaqMan SNP genotyping assay using a predesigned functionally tested probe (Applied Biosystems/Thermo Fisher Scientific) and a 7900HT real-time PCR System (Applied Biosystems). SNP-N-glycome associations were analyzed using an additive genetic model and an FDR of < 0.05.

### Statistics.

Statistical tests and sample numbers are included in figure legends. Unpaired, 1-tailed Student’s *t* test was used for comparison of 2 groups. One-way ANOVA, with Kruskal-Wallis and Dunn’s multiple comparison testing for intergroup comparisons determined by Mann-Whitney test, was used for comparisons of 3 or more groups. Linear regression was used to analyze the weight curves for the DSS experiments. A *P* value of less than 0.05 was considered statistically significant. Note additional statistical considerations for the N-glycome analyses (see above).

### Study approval.

Mouse experiments were approved by the Johns Hopkins University Animal Care and Use Committee. Summary association statistics for rs13107325 and the spectrum of total plasma N-glycome and IgG N-glycome data are publically available (38, 39). Genotype data for the IBD patient cohort analysis were obtained under study approval from Cedars-Sinai Medical Center (no. 3358).

## Author contributions

LS and JM conceived the study. QW, HMN, FW, SM, SW, CS, and JM provided methodology. AF, SS, TH, and JM provided formal analysis. LS, HMN, XW, SW, and JM provided investigation. DM and GL provided resources. JM wrote the original draft of the manuscript. LS, AF, SS, QW, XW, TH, SW, MD, CS, and JM wrote, reviewed, and edited the manuscript. FW, SM, MD, DM, GL, CS, and JM supervised the study. JM acquired funding.

## Supplementary Material

supplemental data

supplemental Table 1

supplemental Table 2

supplemental Table 3

supplemental Table 4

supplemental Table 5

supplemental Table 6

## Figures and Tables

**Table 1:**
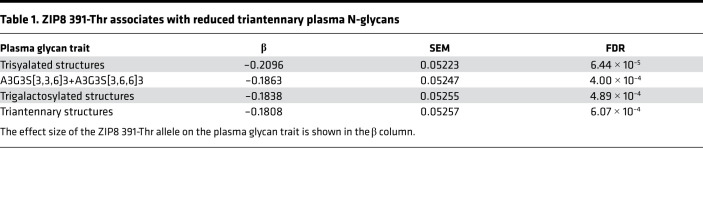
ZIP8 391-Thr associates with reduced triantennary plasma N-glycans

**Table 2:**
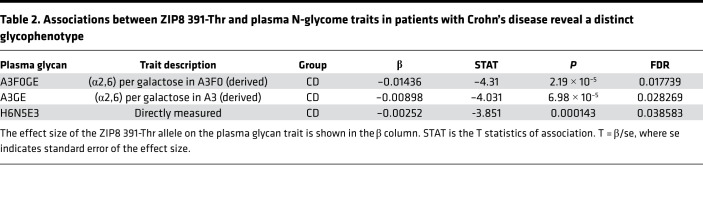
Associations between ZIP8 391-Thr and plasma N-glycome traits in patients with Crohn’s disease reveal a distinct glycophenotype

## Version 1 09/08/2020

In-Press Preview

## Version 2 10/15/2020

Electronic publication
