# Supplementary material for: Pleiotropic ZIP8 A391T implicates abnormal manganese homeostasis in complex human disease
Source: JCI Insight. 2020 Oct 15;5(20):e140978. doi: 10.1172/jci.insight.140978 (PMC7605523; doi:10.1172/jci.insight.140978)
Supplement: supplemental data [file jciinsight-5-140978-s182.pdf]

Supplemental figures

Pleiotropic ZIP8 A391T implicates abnormal manganese homeostasis in complex human disease

Laxmi Sunuwar, Azra Frkatović, Sodbo Sharapov, Qinchuan Wang, Heather Neu, Xinqun Wu, Talin Haritunians, Fengyi Wan, Sarah Michel, Shaoguang Wu, Dermot McGovern, Gordan Lauc, Mark Donowitz, Cynthia Sears, Joanna Melia

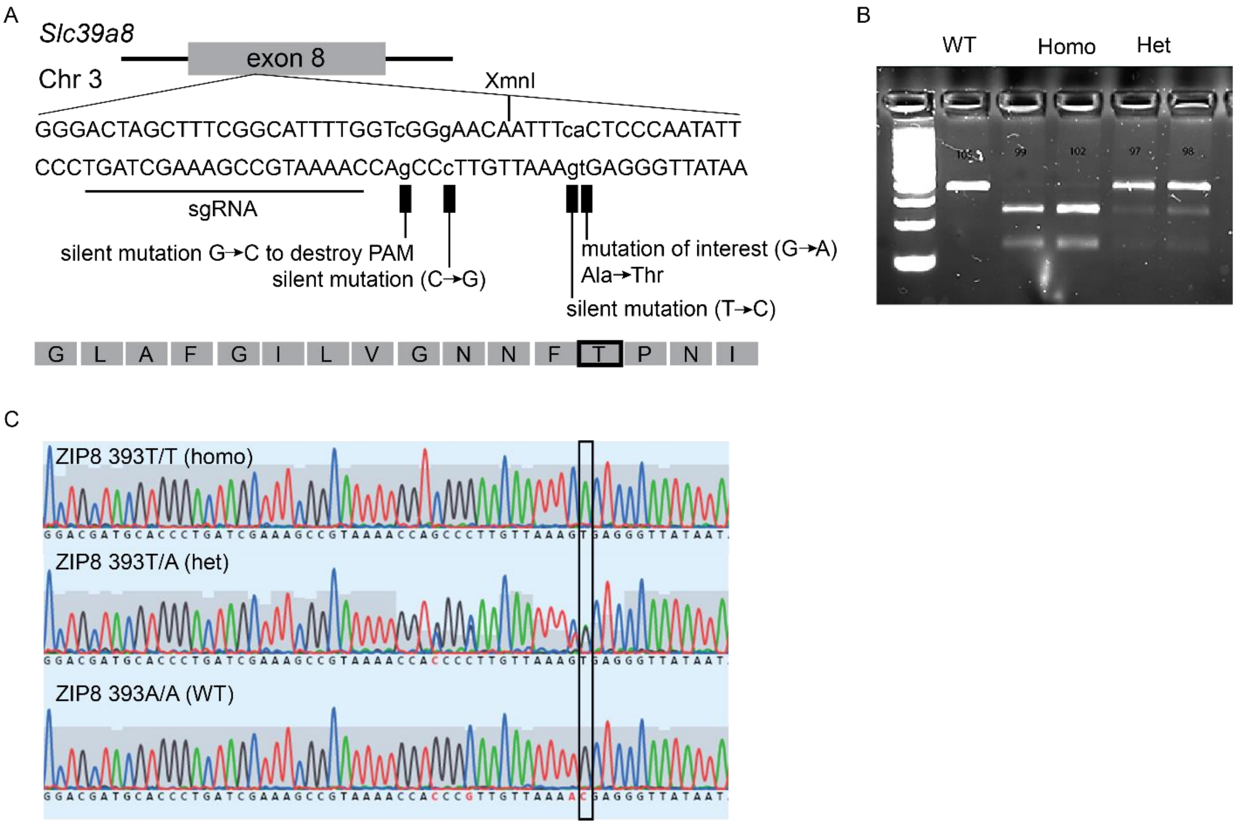

Supplemental Figure 1. Generation of the Zip8 393T-knock-in mouse by CRISPR/Cas9-mediated in vivo gene editing. (A) Short-guide RNAs were designed to target the region of interest. Homologous repair constructs with the mutation of interest and silent point mutations to mutate the PAM site and knock-in restriction enzyme sites to permit genotyping were designed. (B) Genotyping is accomplished by XmnI enzyme digestion after nested PCR with genotyping gel depicted. (C) Genotype confirmed by Sanger sequencing.

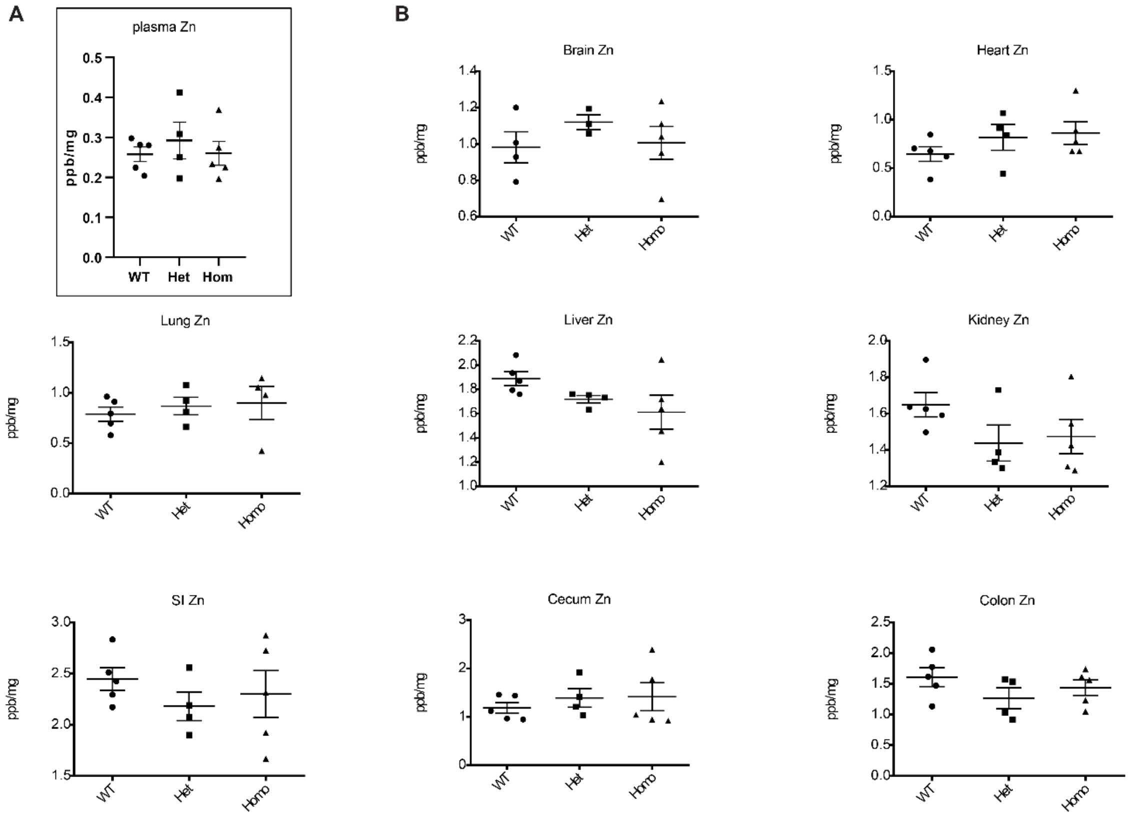

Supplemental Figure 2. Plasma and systemic zinc (Zn) levels in WT and Zip8 393T-KI heterozygous and homozygous male mice. (**A**) Plasma Zn is unchanged across genotypes. (**B**) Differences in systemic Zn levels based on Zip8 393T-KI genotype fails to meet statistical significance, although there is a trend towards reductions in liver and kidney Zn paralleling manganese levels. For all experiments, n=4-5, 8-10 week old male mice. Mean and S.E.M. are graphed. Plasma and tissues were subjected to ICP-MS for metal content, then normalized to wet tissue weight. Statistical analysis by one-way ANOVA with Kruskal-Wallis test when 3 or more groups compared with p-values for intergroup comparisons determined by Mann-Whitney test (S2A-B).

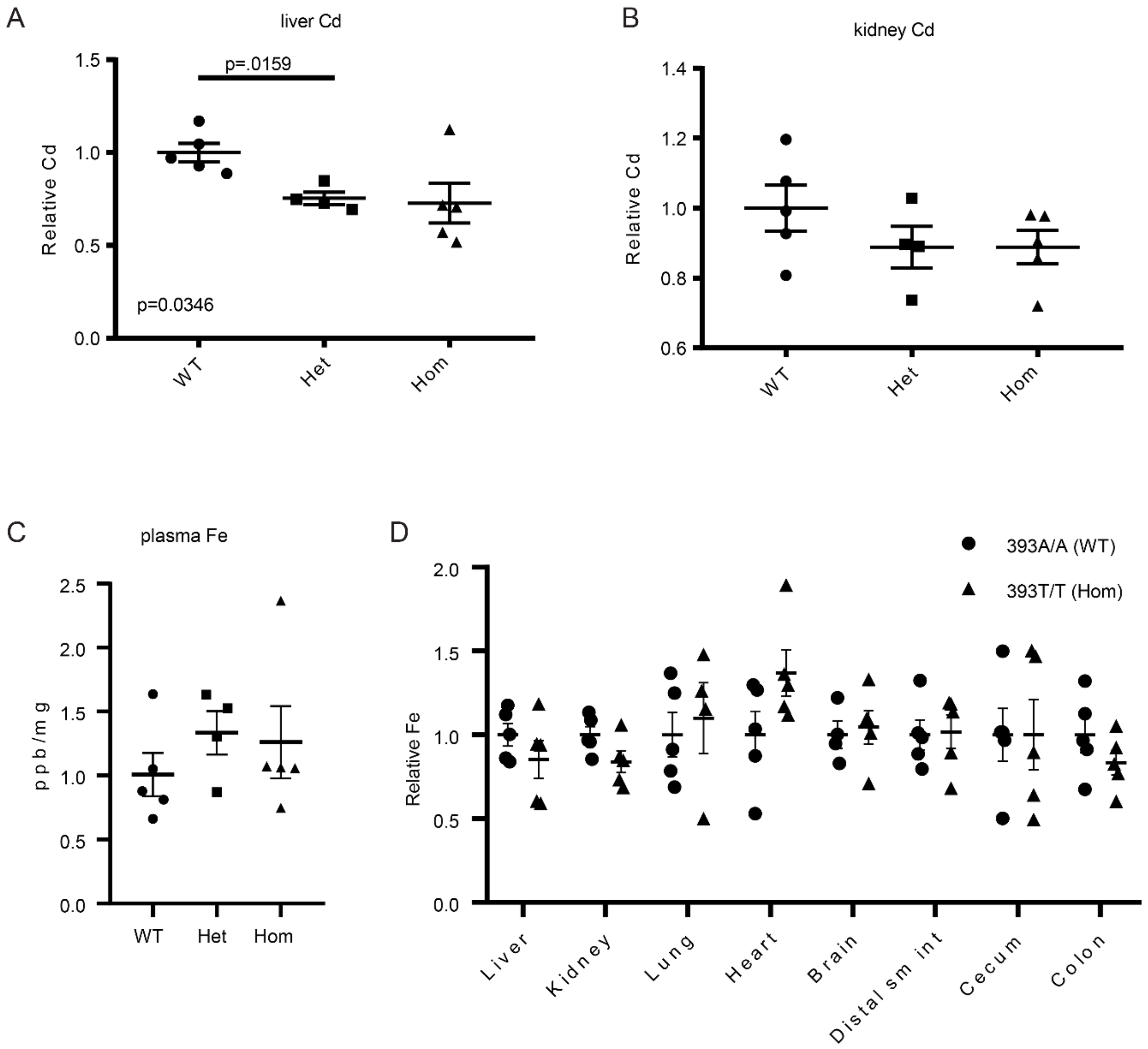

Supplemental Figure 3. Systemic cadmium (Cd) and iron (Fe) levels in WT and Zip8 393T-KI heterozygous and homozygous male mice. (A) Liver Cd is reduced in Zip8 393T-KI heterozygous mice, but fails to meet statistical significance in the homozygous mice. (B) Kidney Cd shows a similar trend to liver CD, but fails to meet statistical significance. (C) Plasma Fe is unchanged across genotypes. (D) Tissue iron is unchanged across genotypes. For all experiments, n=4-5, 8-10 week old male mice. Mean and S.E.M. are graphed. Tissues were subjected to ICP-MS for metal content, then normalized to wet tissue weight. Statistical analysis by one-way ANOVA with Kruskal-Wallis test when 3 or more groups compared with p-values for intergroup comparisons determined by Mann-Whitney test.

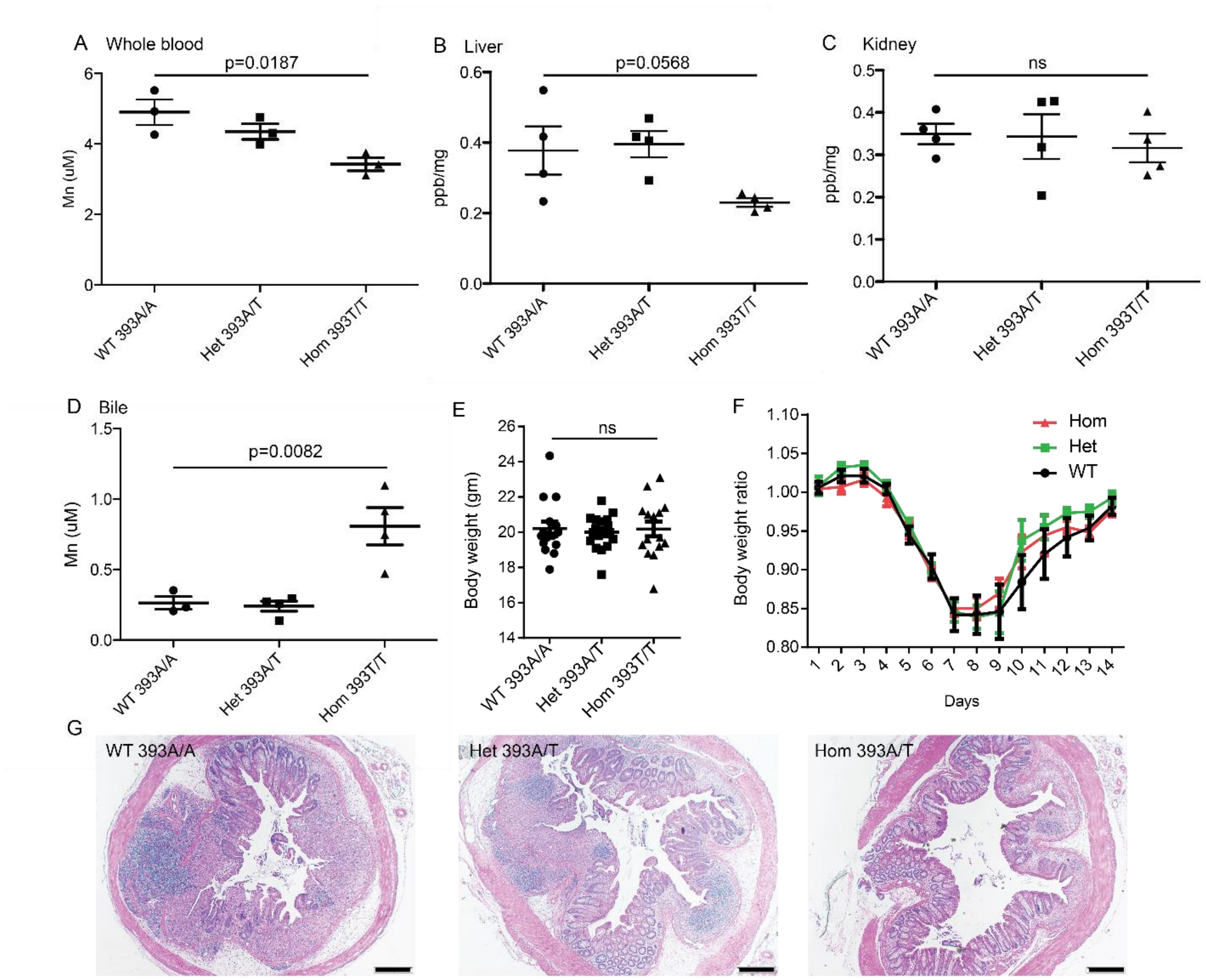

Supplemental Figure 4. Female Zip8 393T-KI homozygous mice exhibit reduced blood Mn and elevated biliary Mn. (**A**) Whole blood Mn is reduced in Zip8 393T-KI homozygous mice (p=0.0187). N=3 female mice/genotype, 8 weeks of age. (**B**) Liver Mn is reduced in female Zip8 393T-KI homozygous mice with nominal significance (p=0.0568). (**C**) Kidney Mn is unchanged between female WT and Zip8 393T-KI mice. N=4 female mice/genotype, 10-12 weeks of age. (**D**) Biliary Mn is increased in female Zip8 393T-KI homozygous mice compared to WT. N=3-4 females/genotype, 8-10 weeks, fasted. (**E**) Initial body weight across genotypes is not different. N=16-20 females/genotype. (**F**) Zip8 393T-KI female mice were not more susceptible to DSS-induced injury than WT mice as measured by body weight loss. (**G**) At Day 14, female mice of all genotypes exhibit ongoing inflammation with areas of ulceration, crypt elongation and crypt distortion. The immune cell infiltrate appears more robust in the WT and heterozygous mice, but this will require further study. H&E images are representative of 3 mice/genotype. Whole blood (Fig. S4A) and bile Mn (Fig. S4D) measured by atomic absorption spectroscopy; tissue Mn measured by ICP-MS and normalized to wet tissue weight (Fig. S4B-C). Mean and S.E.M. are graphed (Fig. S4A-F). Statistical analysis by one-way ANOVA with Kruskal-Wallis and Dunn's multiple comparisons tests when 3 or more groups compared (Fig. S4A-E).

**Supplemental Tables**	
Supplemental Table 1	Normalized differential expression of Nanostring reads for the mouse inflammation codeset. mRNA isolated from the distal colon in mice treated with DSS and sacrificed on Day 8 (Male WT and Zip8 393T-KI homozygous mice)
Supplemental Table 2	Sex-stratified analysis of the association between rs13107325 and IBD, Crohn's disease, and ulcerative colitis
Supplemental Table 3	Plasma and IgG N-glycan analysis of rs13107325 associations
Supplemental Table 4	Plasma and IgG N-glycan analysis of rs13107325 associations in Crohn's disease patients
Supplemental Table 5	Comparison of Mn status across Zip8 inducible knock-out, liver-specific inducible knock-out, and Zip8 393T-KI mouse models
Supplemental Table 6	Primer sequences
