# Supplementary material for: Pleiotropic ZIP8 A391T implicates abnormal manganese homeostasis in complex human disease
Source: JCI Insight. 2020 Oct 15;5(20):e140978. doi: 10.1172/jci.insight.140978 (PMC7605523; doi:10.1172/jci.insight.140978)
Supplement: supplemental Table 5 [file jciinsight-5-140978-s187.pdf]

	Zip8 iKO	Zip8 iKO Het	Zip8 LSKO	Zip8 393T-KI Het	Zip8 393T-KI Hom
Liver	-77%	-39%	-69%	-19%	-28%
Kidney	-52%	-32%	-49%	-17%	-21%
Brain	-31%	-23%	-34%	N.S.	N.S.
Heart	-27%	-14%	-47%	N.S.	N.S.
Small intestine	N.S.	+34%	N.S.	N.S.	N.S.
Whole blood	N.R.	N.R.	-65%	N.S.	-18.9%

Supplemental Table 5. Percent change of tissue and blood Mn in male Zip8 inducible global (iKO) and liver-specific (LSKO)) knockout mice and Zip8 393T-KI heterozygous and homozygous mice compared to age and sex-matched controls. The data for the iKO and LSKO models (12-14 weeks of age) are taken from previous publication (Lin, et al., *JCI*, 2017; PMID 28481222). The Zip8 393T-KI mice were 9-14 weeks for whole blood Mn measurements; 8-10 weeks for tissue measurements. N.R., not reported; N.S., non-significant.
